# High Power Factor of Ag_2_Se/Ag/Nylon Composite Films for Wearable Thermoelectric Devices

**DOI:** 10.3390/nano12234238

**Published:** 2022-11-28

**Authors:** Wenhang Wu, Zheng Liang, Meng Jia, Yuwei Li, Xiongcong Guan, Yunfeng Zhan, Jinxiu Wen, Jianyi Luo

**Affiliations:** 1 Research Center of Flexible Sensing Materials and Devices, School of Applied Physics and Materials, Wuyi University, Jiangmen 529020, China; 2 Guangzhou Institute of Energy Conversion, Chinese Academy of Sciences, Guangzhou 510640, China

**Keywords:** thermoelectricity, Ag_2_Se, Ag, composite film, flexible device

## Abstract

A flexible thermoelectric device has been considered as a competitive candidate for powering wearable electronics. Here, we fabricated an n-type Ag_2_Se/Ag composite film on a flexible nylon substrate using vacuum-assisted filtration and a combination of cold and hot pressing. By optimising the Ag/Se ratio and the sequential addition and reaction time of AA, an excellent power factor of 2277.3 μW∙m^−1^ K^−2^ (corresponding to a *ZT* of ~0.71) at room temperature was achieved. In addition, the Ag_2_Se/Ag composite film exhibits remarkable flexibility, with only 4% loss and 10% loss in electrical conductivity after being bent around a rod of 4 mm radius for 1000 cycles and 2000 cycles, respectively. A seven-leg flexible thermoelectric device assembled with the optimised film demonstrates a voltage of 19 mV and a maximum power output of 3.48 μW (corresponding power density of 35.5 W m^−2^) at a temperature difference of 30 K. This study provides a potential path to design improved flexible TE devices.

## 1. Introduction

Smart wearable devices that can monitor human motion and health conditions are interesting to scientists and engineers [1,2,3]. The majority of wearable devices rely on lithium-ion batteries as their primary power source. However, the battery is an exhaustible source that needs to be replaced or frequently recharged, which limits the lifespan and functionality of wearable devices. Targeting this issue, wearable devices that harvest energy and facilitate the continuous monitoring of data over longer periods have been developed and reported in recent years [4,5,6,7,8]. Energy harvesters can derive their power from a variety of sources, such as vibrations, thermal energy, light, or radio waves [9,10,11,12,13,14]. These energy harvesters can be utilised as an extension to batteries to provide long-term power. Among them, thermal energy harvesting that can convert heat to electricity via the Seebeck effect is a reliable means of direct conversion with no intermediate energy conversion process, compact size operation, and zero pollution [15,16]. Achieving the maximum efficiency of the thermal energy harvester requires an effective way to improve the *ZT* value of thermoelectric (TE) materials. The *ZT* value of a TE material is usually defined as *ZT* = *S*^2^*σT*/*κ*, where *S* is the Seebeck coefficient, which is positive for *p*-type conduction and negative for *n*-type conduction, and *σ* and *κ* represent the electrical conductivity and thermal conductivity, respectively [17]. *T* is the absolute temperature, *S*^2^*σ* denotes the power factor (*PF*), and a high *PF* value means that the device could output a high voltage and current. On the other hand, a high value of *κ* makes it difficult to establish a temperature gradient between the two ends of the material, so even though the *PF* value is constant, the highly thermally conductive material will limit the magnitude of Δ*T* and consequently the electromotive force. A higher *ZT* value means that a material has a high Seebeck coefficient (*S*) and electrical conductivity (*σ*) and a low thermal conductivity (*κ*). TE materials are divided into two categories: organic and inorganic. Organic TE materials, including polypyrrole, polyaniline, and PEDOT:PSS, are composed of carbon-based molecules that present flexibility and low thermal conductivity [18,19,20]. Even though organic TE materials have intriguing properties, their low *ZT* values mean that they are not yet competitive with inorganic TE materials. Inorganic TE materials, such as Bi_2_Te_3_, SnSe, Cu_2_Se, Mg_3_Sb_2_, and Ag_2_Se, have different properties to organic TE materials [21,22,23,24,25,26,27,28,29]. Bi_2_Te_3_ is the best inorganic material for TE applications due to its high *ZT* of 2.4 at room temperature [30]. However, the toxicity of Bi_2_Te_3_ and its poor mechanical properties make it an unsuitable choice for wearable devices [31].

Silver selenide (Ag_2_Se), a new inorganic TE material, is nontoxic and has high electrical conductivity and low thermal conductivity, making it a promising Bi_2_Te_3_ replacement [32,33,34]. The power factor of bulk Ag_2_Se samples that are prepared by the direct reaction of elements Se and Ag increases with temperature, reaching a value of 3500 μW∙m^−1^K^−2^ at 300 K. This trend gives a *ZT* value of 0.96 [35]. By optimising the carrier concentration using a single parabolic band (SPB) model, it is calculated and predicted that the *ZT* value of porous Ag_2_Se can reach approximately 1.1 near room temperature. Additionally, by using the spark plasma sintering (SPS) method to prepare porous Ag_2_Se with hierarchical structures including high-density pores, it was experimentally showed to show *ZT* ~0.9 (*PF* ≈ 2300 μW∙m^−1^K^−2^) at 390 K [36]. The above bulk Ag_2_Se samples exhibit a high *PF* value at room temperature; however, the samples are not flexible. Nevertheless, it is encouraging that a method was recently developed by Professor Cai’s research group to prepare flexible Ag_2_Se/Ag/nylon films. Specifically, Ag_2_Se nanowires are produced by reducing agents reacting with AgNO_3_ to produce Ag nanoparticles, which are then absorbed onto the surface of Se nanowires and react with Se. The Ag_2_Se/Ag/nylon composite films were obtained by vacuum filtration and hot pressing, and the composite films were produced in a *PF* of 1860.6 μW∙m^−1^ K^−2^ at RT (*ZT* ≈ 0.6) [29]. However, the influence of TE properties on the size of Ag particles through the adjustment of the sequence and reaction time of reducing agents AA, as well as the influence of film processing methods, has not been investigated.

In this work, a wet chemical method was utilised to synthesise Ag_2_Se/Ag nanostructures of different sizes. The Ag_2_Se/Ag composite flexible film was then prepared on nylon membranes by vacuum-assisted filtration, followed by combining cold and hot pressing. By optimising the ratio of Ag to Se and the sequence and reaction time of AA addition, an excellent *PF* value of 2277.3 μW∙m^−1^ K^−2^ (a corresponding electrical conductivity of ~2720.1 S∙cm^−1^ and a Seebeck coefficient of ~91.5 μV∙K^−1^) at RT was achieved for the Ag_2_Se/Ag/nylon film. The high electrical conductivity of Ag_2_Se/Ag/nylon composite films leads to their flexibility and lower internal resistance. An optimum film was used to assemble a seven-leg flexible TE device that showed excellent performance. The voltage was observed to be 19 mV and the maximum power output was 3.48 μW (with a corresponding power density of 35.5 W m^−2^) at a temperature difference of 30 K.

## 2. Materials and Methods

### 2.1. Materials

All raw materials, including ethylene glycol (EG, ≥99%), L-ascorbic acid (AA, ≥99%), absolute ethanol (EA), silver nitrate (AgNO_3_, 99.8%), selenium dioxide (SeO_2_, ≥99.99%), and β-cyclodextrin (≥98%) were purchased from Aladdin Co., Ltd. (Shanghai, China). The nylon membrane (aperture: 0.22 μm) was obtained from Jinteng Experimental Equipment Co., Ltd. (Tianjin, China) and the ethanol was of analytical reagent grade. Deionised water was utilised throughout the experiments.

### 2.2. Synthesis of Se NWs

Se nanowires (Se NWs) were synthesised according to the process reported in ref. [28]. Briefly, solution A was prepared by adding 1.0 g SeO_2_ and 1.0 g β-cyclodextrin into 200 mL of deionised water and slowly stirring in a glass beaker. Afterwards, solution B was prepared by adding 2 g of AA into 200 mL of deionised water and slowly stirring in a glass beaker. After complete dissolution, solution A was gradually mixed with solution B while continuously stirring for 4 h at room temperature. The resulting solution was divided into 4 aliquots which were then centrifuged at 4500 rpm for 5 min. The solution was then redispersed into EA solution at the same volume four times. The as-synthesised Se NWs were left to react in 200 mL of EA for 24 h at room temperature. The Se NWs were then centrifuged at 4000 rpm for 5 min. The product was dried in a vacuum at 65 °C overnight and used in the next experiment. The phase composition and morphology of the Se NWs are shown in Figure S1 in the Supplementary Materials.

### 2.3. Synthesis of Ag_2_Se Nanostructures with or without Ag Nanoparticles

Ag_2_Se nanostructures with or without Ag nanoparticles were prepared according to the process reported in refs. [28,29]. Briefly, solution A was prepared by adding 0.1 g of Se NW powder into 28 mL of EG with ultrasonic dispersion in a glass beaker. Then, 0.432 g of AgNO_3_ was added to 56 mL of EG to form solution B, and 0.863 g of AgNO_3_ was added to 113 mL of EG to form solution C. Then, solutions B and C were sonicated and dispersed, respectively. Solution D was prepared by adding 0.223 g of AA into 62 mL of EG with ultrasonic dispersion in a glass beaker. After fully dissolving, solution B was mixed with solution A (named sample 1, S1), and solution C was mixed with solution A (named sample 2, S2). Samples 1 and sample 2 were reacted with continuous agitation at 40 °C for 2 h. For sample 3 (S3), AA was added to sample 2 and then stirred at 40 °C for 3 h. For sample 4 (S4), AA was added to the reaction mixture at 40 °C for 1 h after sample 2 was reacted first for 2 h. The solutions of samples 1–4 were centrifuged at 4000 rpm for 5 min and redispersed into EA solution at the same volume four times. The centrifugal precipitates of the above four samples were dried in a vacuum at 65 °C for 10 h.

The characterisation and measurement of TE properties are also described in Note S1 in the Supplementary Materials.

## 3. Results and Discussion

**Figure 1:**
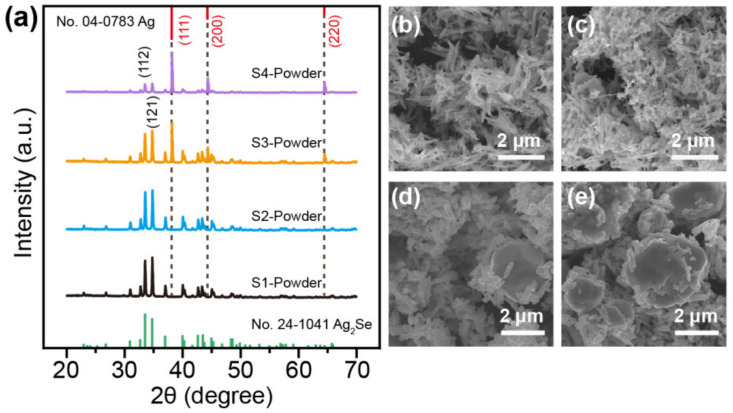
Characterisation of samples 1–4. (**a**) XRD patterns and (**b**–**e**) SEM images of the sample 1–4 powders.

The XRD patterns of prepared samples 1–4 are shown in Figure 1a. All diffraction peaks of the powders of samples 1 and 2 can be indexed to Ag_2_Se (JCPDS No. 24-1041), which indicates that Ag_2_Se produced no significant impurities. Compared with sample 1, sample 2 with a molar ratio of Ag/Se of 4 should produce an excess of silver nanoparticles; however, no silver diffraction peak is observed in the XRD spectrum. The absence of silver peaks can be attributed to either the low concentration of silver nanoparticles or the small size of the silver nanoparticles. This result is attributed to the weak chemical reducibility of EG, which leads to the uniform adsorption of Ag^+^ on the surface of Se NWs. This adsorption, in turn, results in the direct reaction of Ag^+^ with Se NWs, forming spindly Ag_2_Se NWs (Figure 1b,c). If the molar ratio of silver to selenium is greater than 2, too much silver will be present in solution, causing the formation of silver quantum dots or small nanoparticles. Hence, the XRD peaks of the Ag phase are too weak to be detected in sample 2. The chemical reactions of sample 1, which corresponds to Formula (1), and those of sample 2, which corresponds to both Formulas (1) and (2), are shown as follows.

4Ag+ + 2Se + C2H6O2 → 2Ag2Se + C2H2O2 + 4H+(1)

2Ag^+^ + C_2_H_6_O_2_ → 2Ag + C_2_H_4_O_2_ + 2H^+^ (2)

Furthermore, the reducing agent of AA was also utilised in addition to the reducing agent of EG in samples 3 and 4. The difference is that in sample 3, both EG and AA were added to the AgNO_3_ solution and reacted for three hours. In sample 4, however, EG was added to the AgNO_3_ solution and reacted for two hours before being followed by an additional hour of reaction with the AA solution. The XRD patterns of sample 3 and sample 4 reveal the presence of silver (JCPDS No. 04-0783) in addition to Ag_2_Se. This finding is evident from the weaker intensity of the (112) and (121) peaks of Ag_2_Se and the stronger (111) peak of silver. The content of Ag particles in sample 4 appears to be greater than that of Ag_2_Se, as indicated by the stronger XRD peak attributable to the Ag phase. The addition of the strong reductant AA causes the rapid reduction in the dissociated Ag^+^ to Ag nanoparticles. These nanoparticles are then absorbed onto the surface of Se NWs and react to form short Ag_2_Se nanorods. The different reaction mechanisms of sample 3 suggest that it is distinct from sample 2. The chemical reaction formulas are shown in Formulas (3) and (4).

2Ag^+^ + 2C_6_H_8_O_6_ → 2Ag + C_6_H_6_O_6_ + 2H^+^ (3)

2Ag + Se → Ag_2_Se (4)

In sample 4, Ag_2_Se NWs were synthesised in a reductant EG environment for a period, and then the AA reaction was added to rapidly reduce the excessive Ag^+^ into Ag nanoparticles. A small amount of reduced Ag nanoparticles reacted with Se NWs to form Ag_2_Se nanorods. The other large amount of reduced small Ag nanoparticles would tend to agglomerate into large Ag particles in an acidic environment. The primary chemical reaction formulas are referred to as Formulas (1), (3) and (4). The corresponding SEM images for samples 1–4 and energy-dispersive X-ray spectroscopy (EDS) elemental maps of the powders for samples 2–4 are shown in Figure 1b and Figure S2, respectively. The large-grained Ag particles in sample 4 have a size range of 2–10 μm, the smaller Ag particles in sample 3 have a size range of 1–5 μm, and almost no Ag particles were observed in samples 1 and sample 2. Therefore, the size and quantity of Ag particles can be manipulated by changing the reaction time and order of adding AA reductant.

**Figure 2:**
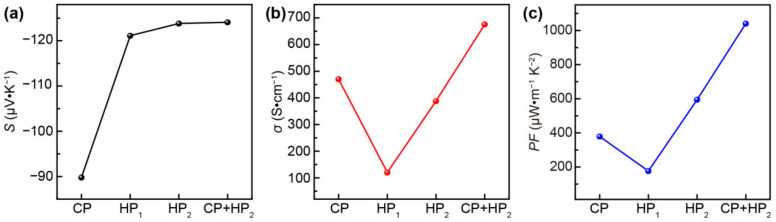
TE performance of Ag_2_Se/nylon film of sample 2 with different film processing methods. (**a**) Seebeck coefficient, (**b**) electrical conductivity, (**c**) power factor.

A simple and effective method to produce 2D wearable TE films with good flexibility is to deposit Ag_2_Se solution onto a porous nylon membrane via vacuum filtration. Nylon film is a flexible substrate with low thermal conductivity and good breathability, making it a suitable substrate for Ag_2_Se TE films with favourable performance and comfort. However, the weak adhesion between Ag_2_Se and nylon with vacuum filtration results in the poor electrical conductivity of the Ag_2_Se TE film. Consequently, it is necessary for an Ag_2_Se film to form a continuous and tight film via high pressure or high temperature. The experimental results of the four samples obtained were found to be similar, with the most significant change seen in sample 2. Herein, the Ag_2_Se/nylon film of sample 2 was selected, and its TE properties were analysed using four film-processing methods. Specifically, the four film processing methods were cold-pressed at 30 MPa (CP), hot-pressed at 100 °C, and 2 MPa (HP_1_), hot-press at 200 °C and 2 MPa (HP_2_), and the combination of cold-press at 30 MPa followed by hot-press at 200 °C and 2 MPa (CP+HP_2_). The TE properties of the Ag_2_Se/nylon film of sample 2 with four film processing methods are shown in Figure 2. Figure 2a shows a negative Seebeck coefficient (*S*), indicating *n*-type conduction. According to the Pisarenko relation, *S* is inversely related to *n* [37]. As a result, the increase in *S* is likely a result of the decrease in *n*. Figure S3a shows that the *n* value decreases from 7.9 × 10^18^ cm^−3^ for the CP film to 1.5 × 10^18^ cm^−3^ for the CP+HP_2_ film. The absolute value of *S* increases from 89.66 μV∙K^−1^ to 123.8 μV∙K^−1^ at RT (Figure 2a). Additionally, the CP film exhibits a dense microstructure with few pores (Figure S3b). The HP_1_ film shows Ag_2_Se NWs sintered into a network-like film with numerous submicron pores (Figure S3c). As a result, the electrical conductivity (*σ*) of the HP_1_ film is the minimum (Figure 2b). In comparison to the HP_1_ film, the HP_2_ film hot pressed at a higher temperature produces a denser film with more distinct grain boundaries (Figure S3d). The surface of CP+HP_2_ film appears to be similar to that of the CP film, with grain boundaries and pores (Figure S3e), which may increase the carrier scattering factor. As shown in Figure 2, the CP+HP_2_ film exhibits the highest *S* absolute value and *σ*, yielding a maximum *PF* value of 1040.3 μW∙m^−1^K^−2^ at RT. The thickness of the films after pressing is illustrated in Figure S4. The thickness from the cross-section is 7 μm for the CP film and the CP+HP2 film, whereas the pure hot-pressed film at a lower pressure is thicker, with a thickness of approximately 20 μm. Noted that if the Ag_2_Se film is directly hot-pressed at 200 °C at a high pressure of 30 MPa, the film will adhere to the platform and damage the surface structure. Therefore, the CP+HP_2_ film processing method is adopted for samples 1–4 to obtain thinner films with better TE performance.

**Figure 3:**
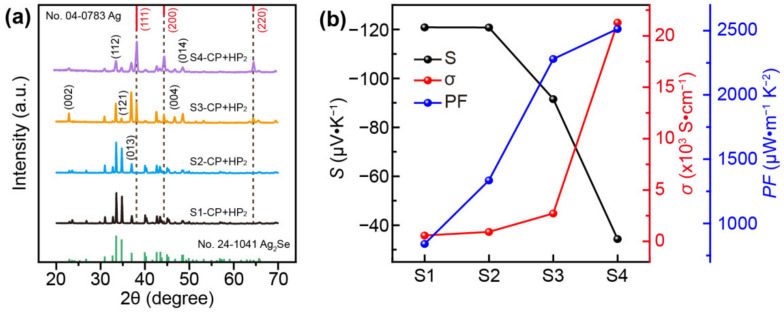
TE performance of Ag_2_Se/nylon films of samples 1–4 under the combination of cold-press and hot-press. (**a**) Seebeck coefficient, electrical conductivity, and power factor. (**b**) XRD patterns of the Ag_2_Se/nylon films corresponding to samples 1–4.

The XRD patterns of four samples deposited on nylon and processed using the CP+HP_2_ procedure are shown in Figure 3a. The diffraction peaks of CP+HP_2_ films of S1 and S2 are similar to those of their corresponding powders. The XRD peaks of S3 and S4 before and after CP+HP_2_ processes show that the two strongest diffraction peaks of (121) and (121) become weak while the (002), (004), (013), and (014) peaks of Ag_2_Se become much stronger after CP+HP_2_ processes. This finding indicates that the Ag_2_Se grains have a preference for growth along the (00*l*) and (01*l*) orientations, which is consistent with previous reports for Ag_2_Se in ref. [28]. The carrier mobility (*μ*) and carrier concentration (*n*) of the Hall measurement for four samples are shown in Figure S5a. From S1 to S4, the value of *n* gradually increases at room temperature, which is inversely proportional to the change in *S*. The absolute value of *S* significantly decreases from 120.9 μV∙K^−1^ for the S1 CP+HP_2_ film to 34.4 μV∙K^−1^ for the S4 CP+HP_2_ film. As shown in Figure 3b and Table S1, the *σ* value dramatically increases from 575.1 S∙cm^−1^ for the S1 CP+HP_2_ film to 21290.1 S∙cm^−1^ for the S4 CP+HP_2_ film. The significant increase in *σ* is attributed to the increased content of highly conducting Ag particles, especially in the S4 CP+HP_2_ film. The Ag phase dominates in this film, as evidenced by the strong (111) peak of the Ag phase (Figure 3a). Hence, the low *S* absolute value of the S4 CP+HP_2_ film is mainly attributed to the high proportion of the Ag phase, which has a rather low *S* absolute value of 5–7 μV∙K^−1^ [35,38,39]. In addition, as there are more Ag particles mixed with Ag_2_Se in S3 and S4, after being cool pressed and then hot pressed, the melted Ag particles converged and dispersed in the Ag_2_Se matrix. The melts then recrystallised when the temperature decreased. The molten Ag particles promoted the sintering of Ag_2_Se and produced numerous opened pores in both the Ag_2_Se phase and Ag phase, which is much different from the pore morphology observed in the S1 and S2 CP+HP_2_ films. In both S3 CP+HP_2_ film and S4 CP+HP_2_ film, there is an obvious energy-filtering effect of the Ag_2_Se/Ag interface. As shown in Figure S6, the internal microstructure of the Ag_2_Se/Ag sample was investigated using HRTEM and EDS mappings. The film contains Ag_2_Se grains of irregular shape and many sub-micron pores (Figure S6a). As shown in Figure S6b,c, the EDS mapping of elements Ag and Se corresponding to Figure S6a reveals that the Ag grain is well combined with the Ag_2_Se phase. Meanwhile, the HRTEM image corresponding to Figure S6d reveals that two inter-planar distances of 0.20 nm and 0.25 nm correspond to the (200) plane of cubic Ag and the (121) plane of orthorhombic Ag_2_Se, respectively. These results suggest that the sample contains both phases of Ag_2_Se and Ag. Therefore, at the interfaces between Ag and Ag_2_Se, the flow of electrons between the two materials creates a potential difference that causes the bands of each material to bend away from the interface (Figure S6e). The potential for this interaction might lead to an energy filtering effect. The high-energy electrons are not influenced by the potential; however, the low-energy electrons are scattered, resulting in an enhancement of *S* [40,41]. On the other hand, the S3 CP+HP_2_ film and S4 CP+HP_2_ film contain pores of submicrometre size, nanograins, and microstructural defects, which are effective in scattering the mid-to-high wavelength phonons and reducing its *κ* value [29]. This finding implies that the TE properties of the Ag_2_Se/Ag film can be further improved by altering the size and quantity of Ag particles, as well as the conductivity and thermal conductivity. As a result, the S3 CP+HP_2_ film and S4 CP+HP_2_ film exhibit extremely high *PF* values of 2277.3 μW∙m^−1^K^−2^ and 2519.4 μW∙m^−1^K^−2^, respectively, at RT. Notably, the value of Ag_2_Se/nylon base films is significantly higher than most previously reported values (Table 1). As the *PF* values are similar, the *ZT* value calculated from the thermal conductivity measurement is another important parameter for evaluating TE properties. As shown in Table S2, the thermal conductivity of the S4 CP+HP_2_ film (1.31 W∙m^−1^K^−1^) is higher than that of the S3 CP+HP_2_ film (0.95 W∙m^−1^K^−1^) due to the increased quantity and size of Ag particles. Hence, the *ZT* value of the S3 CP+HP_2_ film at RT is estimated to be ~0.71, which is approximately 20% greater than the 0.57 *ZT* value of the S4 CP+HP_2_ film. In order to prove the repeatability of the samples, the same set of experiments was also performed in this study, as shown in Figure S7, and the variation trend was basically consistent with Figure 3b.

**Figure 4:**
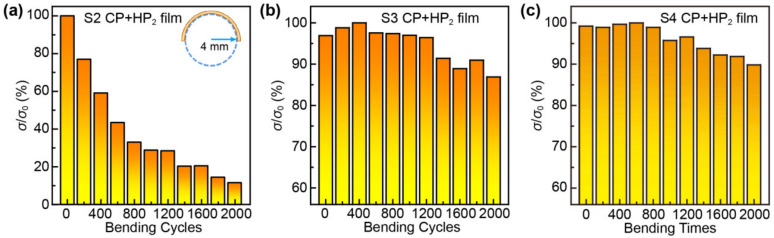
Flexibility property of the Ag_2_Se/nylon film. (**a**–**c**) The ratio of *σ* before and after bending as a function of bending times for the S2, S3 and S4 CP+HP_2_ films, respectively.

Apart from the TE property, the flexibility of TE films is another important factor for practical wearable applications. Hence, the *σ* values of the S2, S3, and S4 CP+HP_2_ films varying with the bending times are measured when the bending radius is 4 mm, as shown in Figure 4. After bending for 1000 and 2000 cycles, the *σ* values of the S3 and S4 CP+HP_2_ film decrease by ~4% and ~10%, respectively. Apparently, the flexibility of S3 and S4 CP+HP_2_ films is apparently superior to that of the *σ* values, which decreases by ~88% after 2000 bending cycles of the S2 CP+HP_2_ film. This phenomenon should be ascribed to the molten Ag film creating a stronger bond between the Ag_2_Se grains and the nylon membrane.

**Figure 5:**
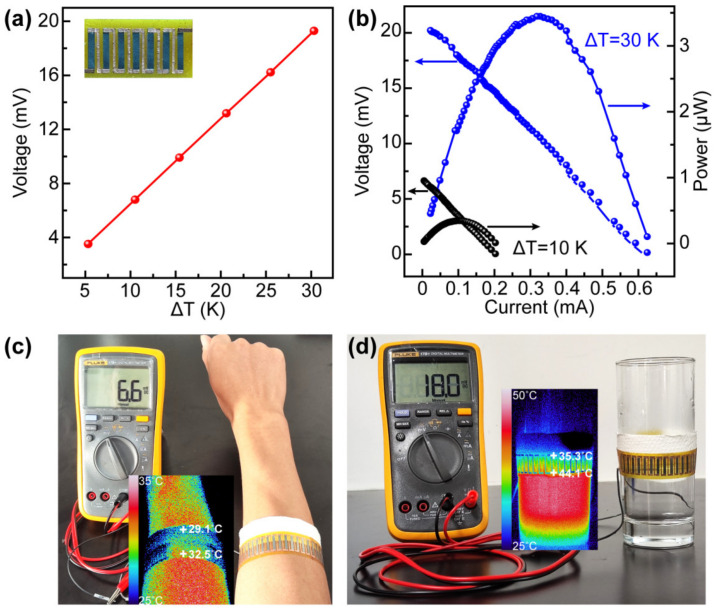
The output performance of the seven-leg TE device fabricated with the S3 CP+HP_2_ film on the PI substrate. (**a**) The relationship between the open-circuit voltage and temperature difference (the inset is a digital photo of the seven-leg TE device). (**b**) The output voltage and power versus current at Δ*T* of 10 K and 30 K. (**c**) A digital photo of 6.6 mV voltage created from the Δ*T* between the arm and the ambient (the inset is the corresponding infrared thermal image of the twenty-three-leg TE device). (**d**) A photo of 18 mV voltage created by the Δ*T* between the upper (cold) and lower (hot) surface of a beaker (the inset is the corresponding infrared thermal image of the twenty-three-leg TE device).

To demonstrate the potential application and to verify the high *PF* of the S3 CP+HP_2_ film, a seven-leg device was fabricated, as illustrated in Figure 5 and the inset of Figure 5a. Each leg with a size of 15 mm × 2 mm × 7 μm was pasted onto a PI substrate. The two ends of each leg were evaporated with a layer of gold to improve contact, and then the legs were attached using a copper paste. Figure 5a shows the relationship between the open-circuit voltage and temperature difference. The open-circuit voltage is directly related to the temperature difference. The measured open-circuit voltage (*U_oc_*) is approximately 19 mV when the temperature difference is 30 K, which is similar to the value calculated according to the expression of *U_oc_* = *N*∙|*S*|·Δ*T* (*N* is the number of TE legs). The output properties of the device were measured by constructing a circuit and adjusting the variable resistance box and temperature of the heating end (Figure S8a). The output voltage (*U_o_*) is inversely proportional to the output current, as shown in Figure 5b. The output power (*P*) of the TE device is expressed as *P* = *U_o_*^2^/(*R*_in_ + *R*_ex_) = *U_o_*·*I*, where *R*_ex_ and *R*_in_ are the external resistance and inner resistance, respectively, of the device. A maximum output power (*P*_max_) is achieved when *R*_ex_ is equal to *R*_in_. By varying the Δ*T*, it was possible to achieve different values of *P*_max_. A Δ*T* of 10 K resulted in a *P*_max_ of 0.36 μW (3.61 mV, 0.10 mA), while a Δ*T* of 30 K resulted in a *P*_max_ of 3.48 μW (10.54 mV, 0.33 mA). This *R*_ex_ value was ~36.1 Ω, whereas *R*_in_ is typically measured to be ~35.6 Ω (Figure S8b). These results show that the *R*_ex_ consisted of two parts: the load resistance (~35.6 Ω) and the internal resistance of the ammeter (~0.5 Ω). On the other hand, *R*_in_ includes the resistance of the TE legs (*R*_1_), electrodes, and the contact resistance between two electrodes of the TE legs (*R*_2_). The measured square resistance of the S3 CP+HP_2_ film (*R*_s_ = *ρ*/*d* = 0.53 Ω/sq) can be used to calculate the resistance of the TE device (*R*_1_ = *N*·*R*_s_·*l*/*w =* 7 × 0.53 × 15 × 10^−3^/(2 × 10^−3^) = 28 Ω). *N*, *ρ*, *d*, *l*, and *w* represent the number of TE devices, resistivity, effective thickness, length, and width of the TE leg, respectively. The resulting resistance, *R*_2_ = *R*_in_ − *R*_1_ = 7.6 Ω, indicates that the resistance of the metal electrodes is negligible. The maximum power density (PD_max_) is calculated by dividing the maximum power (*P*_max_) by the cross-sectional area of the TE leg (*A*). PD_max_ = *P*_max_/(*N*·*A*) = *P*_max_/(*N*·*d*·*w*) = 3.48 × 10^−6^/(7 × 7 × 10^−6^ × 2 × 10^−3^) = 35.5 W·m^−2^ when Δ*T* is 30 K. Herein, the PD_max_ value is powerful and provides enough energy to IoT sensors that typically consume 100 nW–100 mW [42]. Furthermore, as PD_max_ is proportional to Δ*T*^2^/*l*, considering the diversity of Δ*T* and *l* employed in the reported TE devices, it is rational to compare the output performance based on PD_max_ normalised to Δ*T*^2^/*l* (see Table 1). Notably, the value of PD_max_·*l*/Δ*T*^2^ in the present work is higher than that of previously reported flexible TE devices. In Figure 5c, the twenty-three-leg TE device is wrapped around the arm with one side in direct contact with the skin and the other side isolated from the skin by insulating paper. A temperature difference of approximately 3.4 K between the arm and the environment generates a voltage of 6.6 mV (the temperature is measured with an infrared thermometer). The twenty-three-leg TE device from Figure 5d is wrapped in a beaker containing hot water. A voltage of 18 mV is generated from a temperature difference of 8.8 K between the upper surface (cold) and the lower surface (hot). This result indicates that Ag_2_Se/Ag/nylon films have great potential for the application of wearable devices due to their favourable properties.

**Table 1:** Comparison of TE performance of Ag_2_Se/nylon-based films at room temperature.

Material	*S* (μV·K^−1^)	*σ* (S·cm^−1^)	*PF* (μW·m^−1^ K^−2^)	PD_max_ *l*/Δ*T*^2^ (μW m^−1^ K^−2^)	Ref.
Ag_2_Se/Nylon	−140	497	987	51.1	[28]
Ag_2_Se/Nylon	−143	920	1882	488.9	[43]
Ag_2_Se/Ag/CuAgSe/Nylon	−45.5	10770	2232	75.57	[39]
Ag_2_Se/Ag/PEDOT/Nylon	−49.2	5957.3	1443	204	[44]
Ag_2_Se/SWCNTs/Nylon	−121	704	1031	/	[45]
Ag_2_Se/Ag/Nylon	−70	3958	1861	239.8	[29]
Ag_2_Se/Ag/Nylon	−91.5	2720.1	2277.3	591.7	This work

## 4. Conclusions

A flexible Ag_2_Se/Ag composite film on nylon substrate was successfully prepared via facile wet chemical synthesis followed by vacuum-assisted filtration and a combination of cold and hot pressing. The optimised film (with an Ag/Se molar ratio of 4:1 and AA simultaneously added to reactions to react for 3 h) exhibits an excellent *PF* of 2277.3 μW·m^−1^ K^−2^ at room temperature, mainly originating from the combined effect of the Ag and Ag_2_Se phases. In addition, the optimum film also exhibits outstanding flexibility. A seven-leg TE device assembled with the optimised film generates a voltage of 19 mV and a maximum power of 3.48 μW (corresponding power density of ~35.5 W m^−2^) at a temperature difference of 30 K. This work offers a successful approach to creating high-performance and flexible Ag_2_Se-based TE composite films, which can be extended to other TE film systems.

## Data Availability

The data are available within the manuscript and the corresponding Supplementary Materials File.

## Supplementary Materials

The following supporting information can be downloaded at: https://www.mdpi.com/article/10.3390/nano12234238/s1, Note S1: Measurement and characterisation; Note S1: Measuring principle of the in-plane thermal conductivity; Figure S1: Characterisation of Se NWs; Figure S2: Microstructure characterisation of the powder of sample 2, sample 3, and sample 4; Figure S3: Characterisation of Ag2Se/nylon film of sample 2 with different film processing methods; Figure S4: Cross-section SEM images of Ag2Se/nylon film; Figure S5: The carrier behaviours and morphology characterisation of sample 1–4; Figure S6: The microstructure of the Ag2Se/Ag sample; Figure S7: TE performance of Ag2Se/nylon films of another set of samples 1–4 under the combination of cold-press and hot-press; Figure S8: The electrical properties of the TE device; Figure S9: Schematic diagram of the in-plane mode of laser flash measurement. Table S1: Thermoelectric properties of the four samples at room temperature; Table S2: Details of in-plane thermal conductivity measurement of the Ag2Se/Ag/nylon films of S3 and S4.
