# Supplementary material for: High Power Factor of Ag2Se/Ag/Nylon Composite Films for Wearable Thermoelectric Devices
Source: Nanomaterials (Basel). 2022 Nov 28;12(23):4238. doi: 10.3390/nano12234238 (PMC9737951; doi:10.3390/nano12234238)
Supplement: Supplementary file 1 [file nanomaterials-12-04238-s001.zip › nanomaterials-1963614-supplementary.pdf]

nanomaterials

Supplementary Materials

High Power Factor of Ag_2Se/Ag/Nylon Composite Films for Wearable Thermoelectric Devices

Wenhang Wu [1], Zheng Liang [2], Meng Jia [1], Yuwei Li [1], Xiongcong Guan [1], Yunfeng Zhan [1], Jinxiu Wen [1,*] and Jianyi Luo [1,*]

[1] Research Center of Flexible Sensing Materials and Devices, School of Applied Physics and Materials, Wuyi University, Jiangmen 529020, China
[2] Guangzhou Institute of Energy Conversion, Chinese Academy of Sciences, Guangzhou 510640, China
* Correspondence: jinxiuwen@foxmail.com (J.W.); luojiany@mail3.sysu.edu.cn (J.L.)

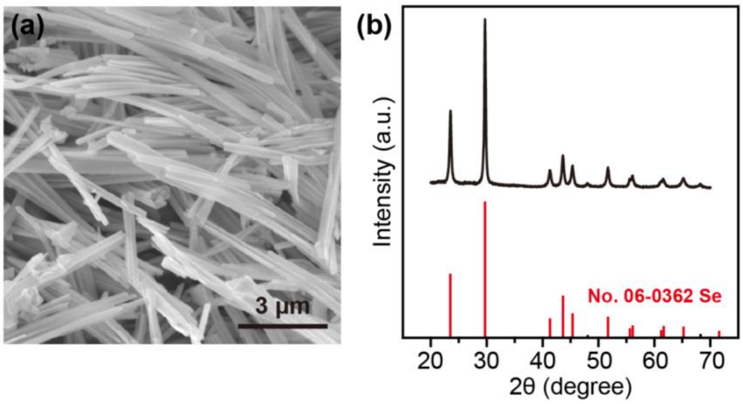

Figure S1. Characterization of Se NWs. (**a**) SEM image of Se NWs, (**b**) XRD pattern of Se NWs.

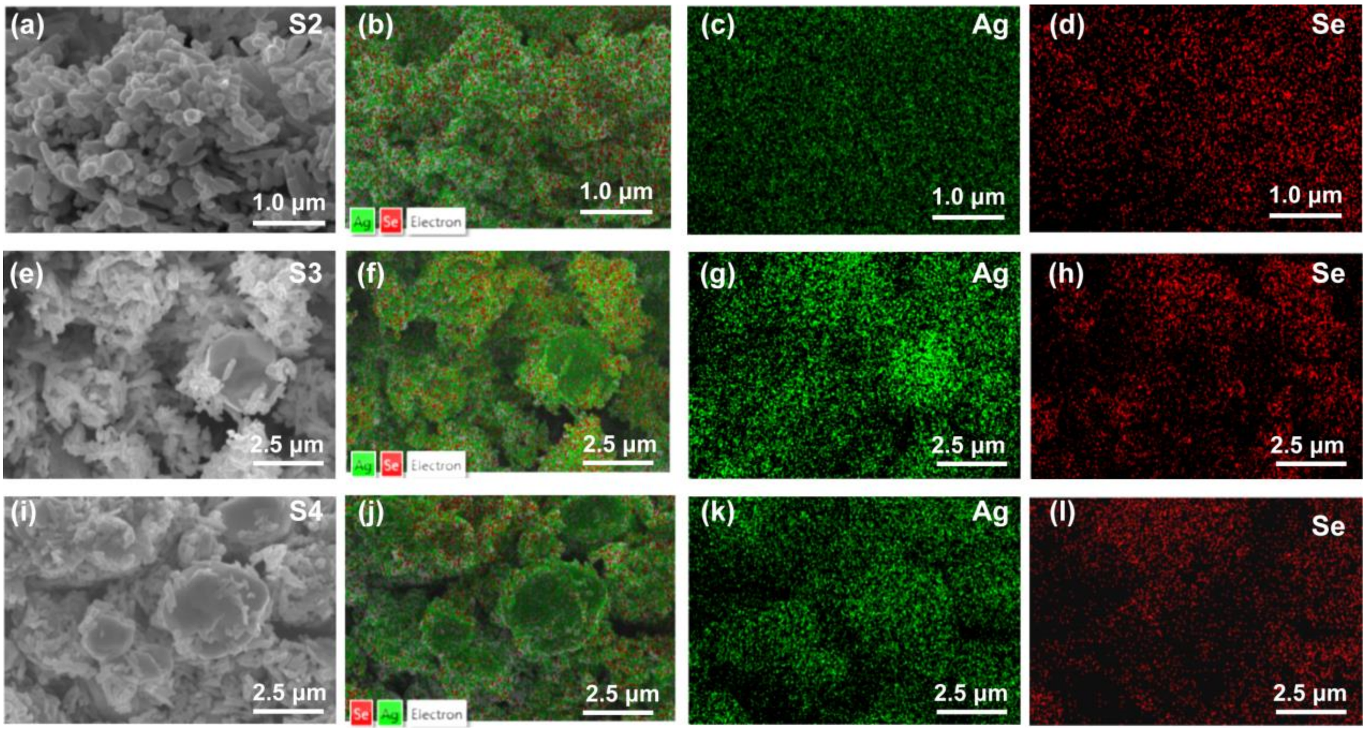

Figure S2. Microstructure characterization of the powder of sample 2, sample 3 and sample 4. SEM images of sample 2 (**a**), sample 3 (**e**) and sample 4 (**i**). EDS mappings of element Ag (**c**, **g**, **k**), Se (**d**, **h**, **l**) and the elements combined ESD mappings (**b**, **f**, **j**) corresponding to S2, S3 and S4.

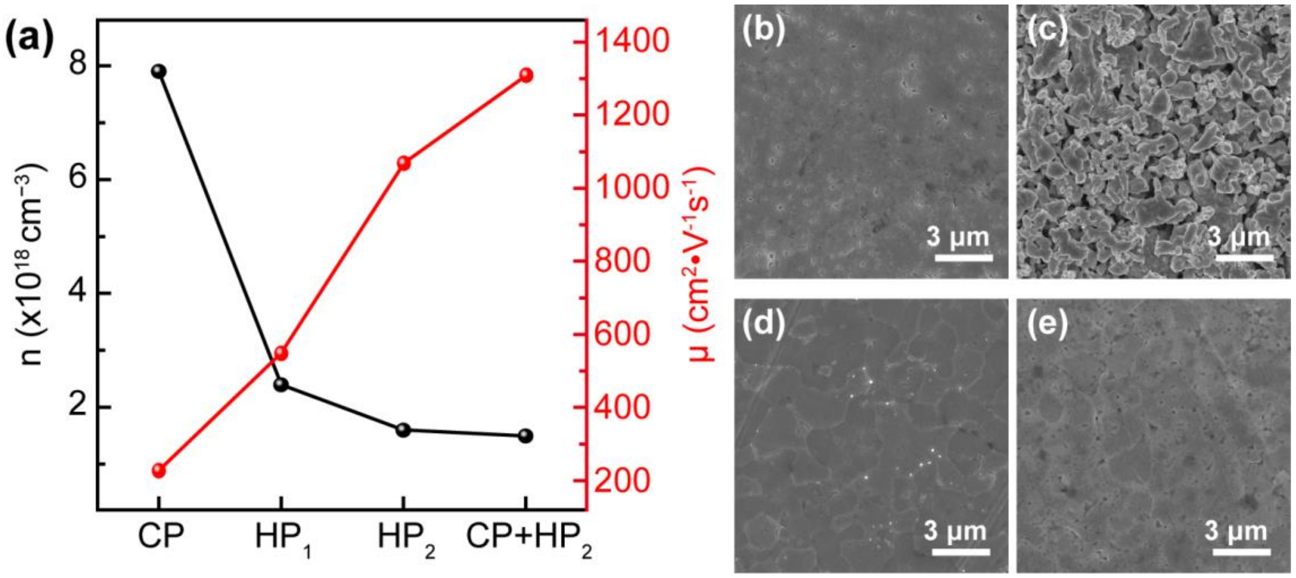

Figure S3. Characterization of the Ag_2Se/nylon film of sample 2 with different film processing methods. The carrier concentration and mobility (**a**) and SEM images of the film processing conditions of cold-press at 30 MPa (**b**), hot-press at 100 °C (**c**) and 200 °C (**d**), and the combination of cold-press at 30 MPa and hot-press at 200 °C (**e**), respectively.

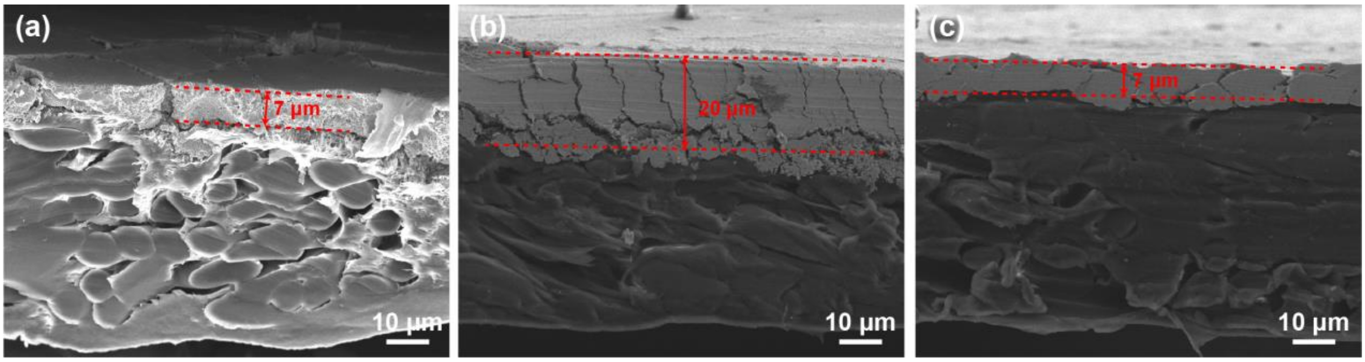

Figure S4. Cross-section SEM images of Ag_2Se/nylon film. The film processing conditions of the cold-pressed at 30 MPa (**a**), hot-press at 200 °C (**b**), and the combination of the cold-press at 30 MPa and hot-press at 200 °C (**c**), respectively.

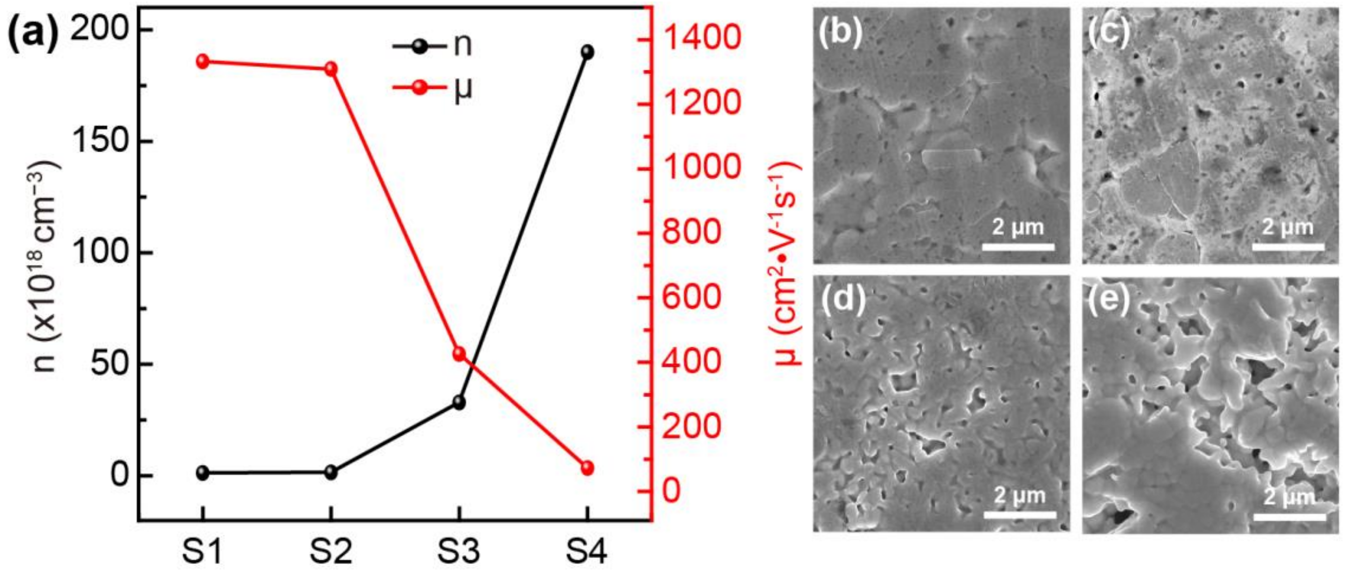

Figure S5. The carrier behaviours and morphology characterization of samples 1–4. Carrier concentration and mobility (**a**), and surface SEM images (**b**–**e**) of the Ag_2Se/nylon films corresponding to samples 1–4 under the combination of cold-press and hot-press.

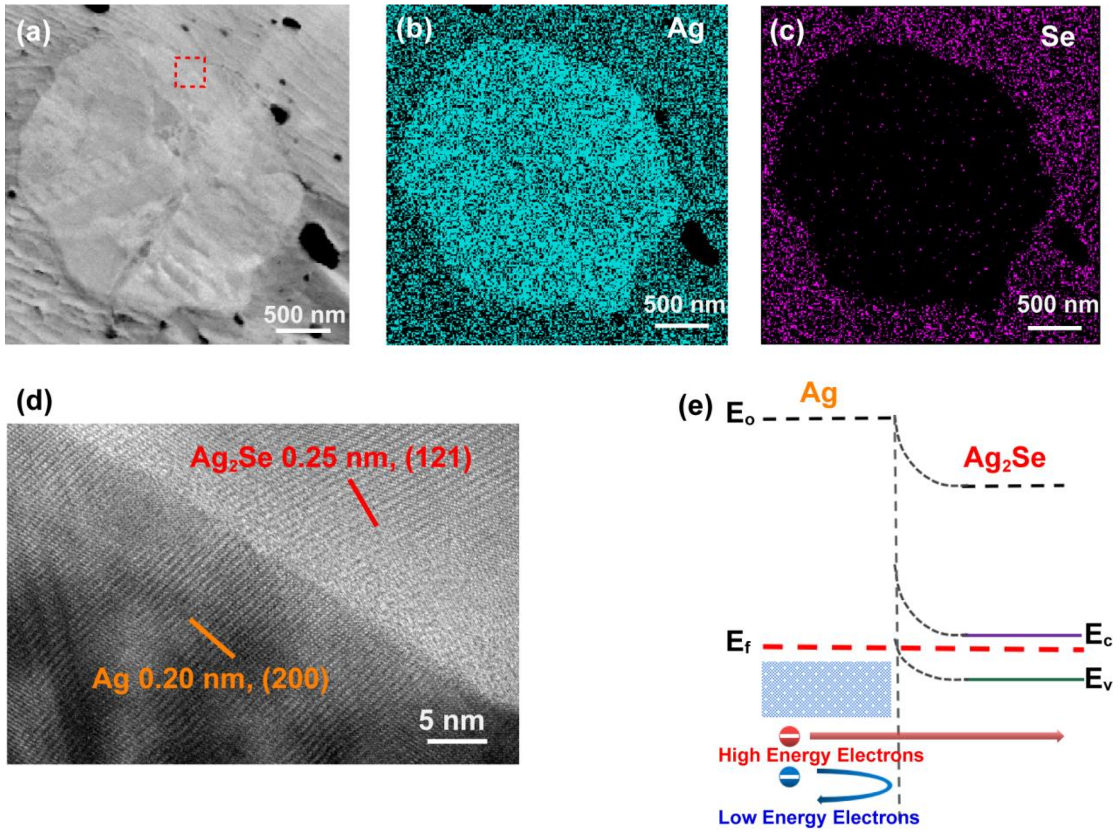

Figure S6. The microstructure of the Ag_2Se/Ag sample. (**a**) TEM image of the interface between Ag_2Se and Ag phases. (**b**, **c**) the EDS mappings of elements Ag, Se corresponding to the red square marked in (**a**). (**d**) HRTEM image of the red rectangle marked in (**a**). (**e**) The energy band diagram for Ag_2Se/Ag sample.

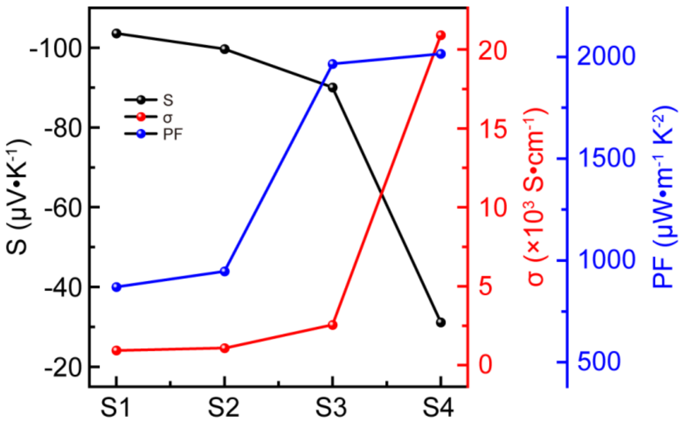

Figure S7. TE performance of Ag_2Se/nylon films of another set of samples 1−4 under the combination of cold-press and hot-press.

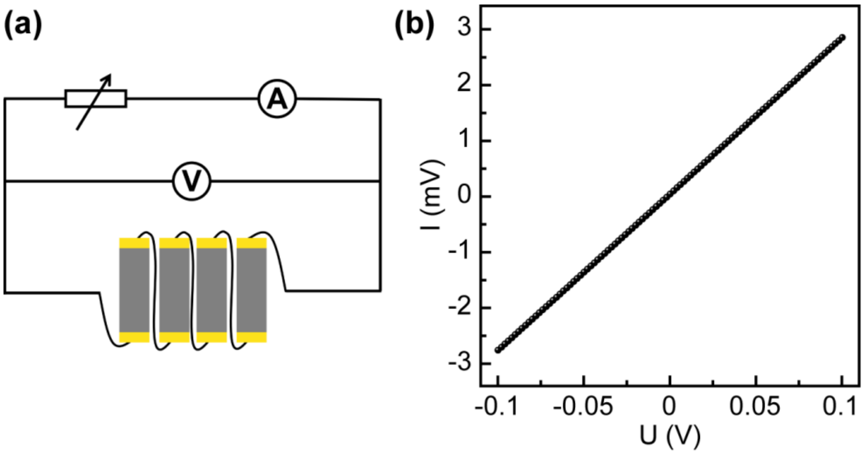

Figure S8. The electrical properties of the TE device. (**a**) Illustration of the TE device output performance measurement. (**b**) The IV curve of the seven-leg TE device.

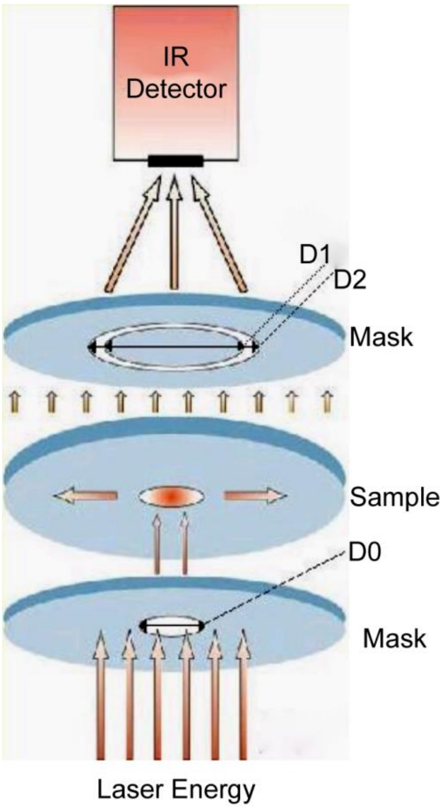

Figure S9. Schematic diagram of the In-plane mode of laser flash measurement.

Table S1. Thermoelectric properties of the four samples at room temperature.

Sample	S (μV·K^{-1})	σ (S·cm^{-1})	PF (μW·m^{-1}K^{-2})
S1 CP+HP_2 film	−120.9	575.1	840.6
S2 CP+HP_2 film	−120.8	914.4	1334.4
S3 CP+HP_2 film	−91.5	2720.1	2277.3
S4 CP+HP_2 film	−34.4	21,290.2	2519.4

Table S2. Details of in-plane thermal conductivity measurement of the Ag_2Se/Ag/nylon films of S3 and S4.

Sample	C_p (J·g^{-1}K^{-1})	ρ (g·cm^{-3})	D (mm^2·s^{-1})	κ (W·m^{-1}K^{-1})
S3 CP+HP_2	0.890	1.689	0.63	0.95
S4 CP+HP_2	0.730	1.679	1.07	1.31

Note S1

Measurement and Characterization

The temperature dependence of S of the films was tested by a thermoelectric test system (TLTP-TEC2415D-WF100, Wuhan, China). The Seebeck coefficient (S) at RT was determined by the slope of the linear relationship between the thermal electromotive force and temperature difference (ΔT) between the two ends of each film. The electrical conductivity (σ) at RT was measured using a four-probe technique (RST-9, Guangzhou, China). The carrier concentration (n) and mobility (μ) were measured by the Van der Pauw method (HMS-5000, Seoul, South Korea). The in-plane thermal conductivity κ was measured by the laser flash method (LFA 467 HyperFlash, Munich, Germany), and specific heat capacity Cp was evaluated by the differential scanning calorimetry (METTLER DSC1, Zurich, Switzerland). The density ρ was tested by measuring the mass and geometrical dimensions of the film with nylon ($\rho = m/V$). The mass (m) is weighed by a balance and

the volume is calculated by measuring the diameter (*D*) and thickness (*H*) of the film, the formula as $V = \pi D^2 H/2$. The phase composition of Ag_2Se nanostructures, Ag particles and Se NWs was measured by X-ray diffraction (XRD, X'Pert PRO, Almelo, Netherlands) at room temperature with Cu Kα radiation at 40 kV and 40 mA. The test range of XRD patterns was $10° \leq 2\theta \leq 70°$. The surface morphology and thicknesses of these composite films were observed by the field emission scanning electron microscope (SEM, Sigma 500, Oberkochen, Germany) with an accelerating voltage of 15 kV. Simultaneously, the energy spectrum analysis was measured by X-ray energy dispersive spectroscopy (EDS, X-MAX 50, London, England).

Measuring principle of the in-plane thermal conductivity

A beam of laser energy irradiates a small central region on the lower surface of the sample. This energy is then transmitted inside the sample in two directions, either vertically or horizontally. The energy in the central region directly transmitted in the vertical direction will be blocked by the cover of the sample bracket above the sample and cannot be detected by the infrared detector. After a period of conduction, the energy transmitted in the horizontal direction finally comes out through the hollow area of the cover of the sample bracket and is detected by the infrared detector. Therefore, the signal detected by the infrared detector is the part of the laser energy after a distance of plane conduction (the influence of vertical conduction can be corrected by the model). The thermal diffusion coefficient in the plane direction of the material can be calculated by fitting the measurement results with the unique in-plane mathematical model. After instrument model modification, the horizontal thermal conductivity can be approximately obtained (Figure S9).
